# Modeling heterogeneity, commitment, and memory of bacterial spore germination

**DOI:** 10.1128/mbio.00596-25

**Published:** 2025-04-02

**Authors:** William Li, Steven Mednick, Peter Setlow, Yong-Qing Li

**Affiliations:** 1 Marshall School of Business, University of Southern California 33902, Los Angeles, California, USA; 2 Department of Molecular Biology and Biophysics, UConn Health 705913, Farmington, Connecticut, USA; 3 Department of Physics, East Carolina University 169119, Greenville, North Carolina, USA; Washington University School of Medicine, Saint Louis, Missouri, USA

**Keywords:** bacterial spore, spore germination, cellular heterogeneity, spore memory, mathematical modeling

## Abstract

Spores of bacteria are metabolically dormant, resistant to microbicides, and vectors of food spoilage and diseases and survive for years in their dormant state. Upon exposure to nutrient germinants, spores can rapidly return to life through germination, losing their resistance and becoming easy to kill. Despite extensive research on germination heterogeneity, commitment, and memory, many mechanisms underlying germination of Bacillota spores remain unclear, as a comprehensive mathematical model describing germination characteristics of individual spores is lacking. Woese et al. (PNAS, 59:869, 1968) developed a simple model predicting that time-to-germination of a spore with *n* active enzymes is proportional to 1/*n*. Here, we present a novel approach inspired by artificial neural networks to model spore germination, treating it as a decision-making process upon the activation produced by binding of germinants to germinant receptors. Major findings include the following: (i) using a sigmoid activation function to model germination thresholds allows predictions of distributions in time-to-commitment and kinetic germination to be well fitted to experimental observations; (ii) modeling spore commitment and memory after two separate germinant pulses fits well to experimental data of *Bacillus* spores germinated with L-alanine, and a zero fraction of germinated spores by a second pulse is predicted by loss of memory; and (iii) modeling kinetic CaDPA release from individual spores through SpoVA channels and fitting experimental data of *Bacillus cereus* spores. This work enhances our understanding of unexplored biophysical intricacies of spore germination, and the use of the model may generate new data.

**IMPORTANCE:**

Spore germination is a crucial process through which spores of bacteria return to life when triggered by germinants, and some spore species cause food spoilage, human diseases, and bioterrorism. Understanding and theoretical predictions of spore germination could facilitate the development of “germinate to kill” strategies as spores lose their resistance upon germination. Here, we developed a novel mathematical model to describe the characteristics of spore germination including heterogeneity, commitment, memory, and kinetic CaDPA release using an artificial neural network. This model predicts new aspects of germination such as the retention and loss of memory and the effect of GRs’ distribution on germination rate and could be useful in data-driven discoveries to enhance our understanding of germination’s biophysical intricacies.

## INTRODUCTION

Spores of bacteria are dormant and resistant to multiple stresses including heat, radiation, and chemicals (1, 2). Consequently, spores survive for years without nutrients but can be vectors for food spoilage and serious diseases such as anthrax. Importantly, spores can sense and respond to external stimuli through germinant receptors (GRs), and when nutrient germinants become available, spores return to life in germination, lose their resistance properties, and become easy to kill (3, 4).

Although not fully understood, much has been learned about germination of spores of Bacillota (5–8). Germination is commonly triggered by spores’ sensing small molecule germinants by GRs (1, 2), with spores of *Bacillus* species containing multiple specific GRs in their inner membrane (IM). When appropriate germinants are present, these compounds activate specific GRs and trigger germination, and spores release monovalent cations, followed by release of the spore core’s depot of dipicolinic acid in a 1:1 complex with Ca^2+^ (CaDPA, ~20% of core dry wt). *Bacillus subtilis* spores contain three functional GRs, GerA, GerB, and GerK. GerA responds to L-alanine or L-valine (L-val), and GerB and GerK are both required for germination with an L-asparagine (L-asn), D-glucose, D-fructose, and K^+^ (AGFK) mixture. After germinant exposure, GR’s germination signal is transduced and amplified, activating downstream components of the germination apparatus, particularly SpoVA proteins. These proteins make up a crucial spore IM channel, and its activation triggers the release of the spore core’s CaDPA depot.

Large amounts of data on spore germination have been gathered from both spore populations and individuals with key features including heterogeneity, commitment, and memory (9–18). Heterogeneity denotes the variability among individual spores in the time between addition of germinants and initiation of rapid CaDPA release (9–13). This heterogeneity is due in part to variations in numbers of GRs between individual spores, with spores having more GRs germinating faster than those with fewer (14). Additionally, mutant spores with elevated GR levels germinate faster than wild-type spores (11). Spore’s commitment is the phenomenon that exposure of spores to a short germinant pulse can result in a fraction of spores committing to germinate even if the germinants are removed or their binding to the GRs is blocked (15–17). The cascade of events after the irreversible commitment step generally includes (i) changes in the permeability of the spore IM, (ii) release of monovalent cations and the core’s CaDPA depot, (iii) CaDPA’s replacement by water, and (iv) hydrolysis of the spore’s peptidoglycan cortex. The spores not committed to germinate carry out no germination events.

Spore memory refers to the phenomenon whereby spores retain a cellular response to an initial transient exposure to germinant stimuli, such that a second exposure triggers even more efficient germination. This was first observed by Zhang et al. (17), where *B. subtilis* spores of PS3415 having high levels of the GerB* GR that responds to L-asn alone exhibited a much higher germination response to a second pulse of L-asn. It appeared that the first pulse induced a change in the spores that did not germinate during the first exposure, enabling them to retain a response to the second pulse. Further observations indicated that the memory of the first pulse decayed over time, especially if there was a longer interval between the pulses or if the spores were incubated at a high temperature (17, 18). Spore memory was also confirmed in *B. subtilis* spores with different nutrient-triggered GRs and in spores of other *Bacillus* species and *Clostridium* (18). Recent studies re-examined this phenomenon in *B. subtilis* spores germinating with L-alanine (6, 7). It has been proposed that spore memory is primarily stored in metastable states of SpoVA proteins (18), and/or that the spore’s electrochemical potential plays a role in enabling this memory (6). However, the involvement of electrochemical potential in the memory of germinant exposure appears very unlikely (7). Consequently, the exact mechanism of spore memory remains not fully understood.

Modeling spore germination has been extensively explored (19–22), but a comprehensive mathematical model that explains spore heterogeneity, commitment, and memory is still lacking. The pioneering work on modeling germination kinetics was conducted by Woese et al. (19) in 1968, in which germination was assumed to result from the accumulation of a substance *P*, with its production rate being proportional to the number of activated germination “enzymes.” By assuming that a threshold amount of *P* was required for germination, it was found that the time needed for a spore with n active enzymes to germinate was proportional to 1/n. Furthermore, by assuming that the fraction of spores with n active enzymes in a population follows a Poisson distribution, this simple model explained spore heterogeneity and germination kinetics, which exhibited a step-like behavior but was not consistent with the experimental data (19). Indest et al. (20) highlighted molecular mechanisms of spore germination, and a mathematical model was elaborated in (14). Peleg and Normand (21) developed models under static and dynamic conditions to predict spore germination behavior, employing both empirical and mechanistic approaches. These studies have shown that while existing models can capture certain aspects of spore germination, they fail to fully explain the observed phenomena of heterogeneity, commitment, and memory.

In this work, we develop a comprehensive mathematical model to describe characteristics of spore germination including heterogeneity, commitment, memory, and kinetic CaDPA release inspired by an artificial neural network (ANN). ANNs are a cornerstone of computational neuroscience and machine learning (23, 24), which have become a powerful tool for wide applications, from image and speech recognition to predictive modeling and decision-making systems. An ANN model consists of connected nodes called artificial neurons. Each neuron in the input layer receives an input signal *x*_*i*_, weighted by *g*_*i*_, and then summed and biased to send a signal *z* to activate a function *f*(*z*) to generate an output y (Fig. S1A). A sigmoid activation function is commonly used in ANNs to aid the training of the network due to its smooth gradient compared to the step function (25). We observe a similarity in decision-making processes between spore germination and the ANN algorithm, suggesting that ANN algorithms can be utilized for modeling the complex process of spore germination. We describe GRs’ activation by binding with germinants and production of a germination trigger signal *z* by the activated GRs, which act as the activated germination “enzymes” and germination substance *P* in Woese et al.’s (19) model. However, we use a sigmoid activation function instead of a step function for modeling germination threshold, which predicts smooth kinetic germination distributions without the step-like behavior and fits well to the experimental data. Our model can explain new aspects of spore heterogeneity, commitment, memory, and kinetic CaDPA release, which can be used to test its validation and/or enhance our understanding of biophysical intricacies of the process. The model could be useful in generating new data in several areas and enables data-driven discoveries.

## RESULTS

### Theoretical model and assumptions

**Fig 1:**
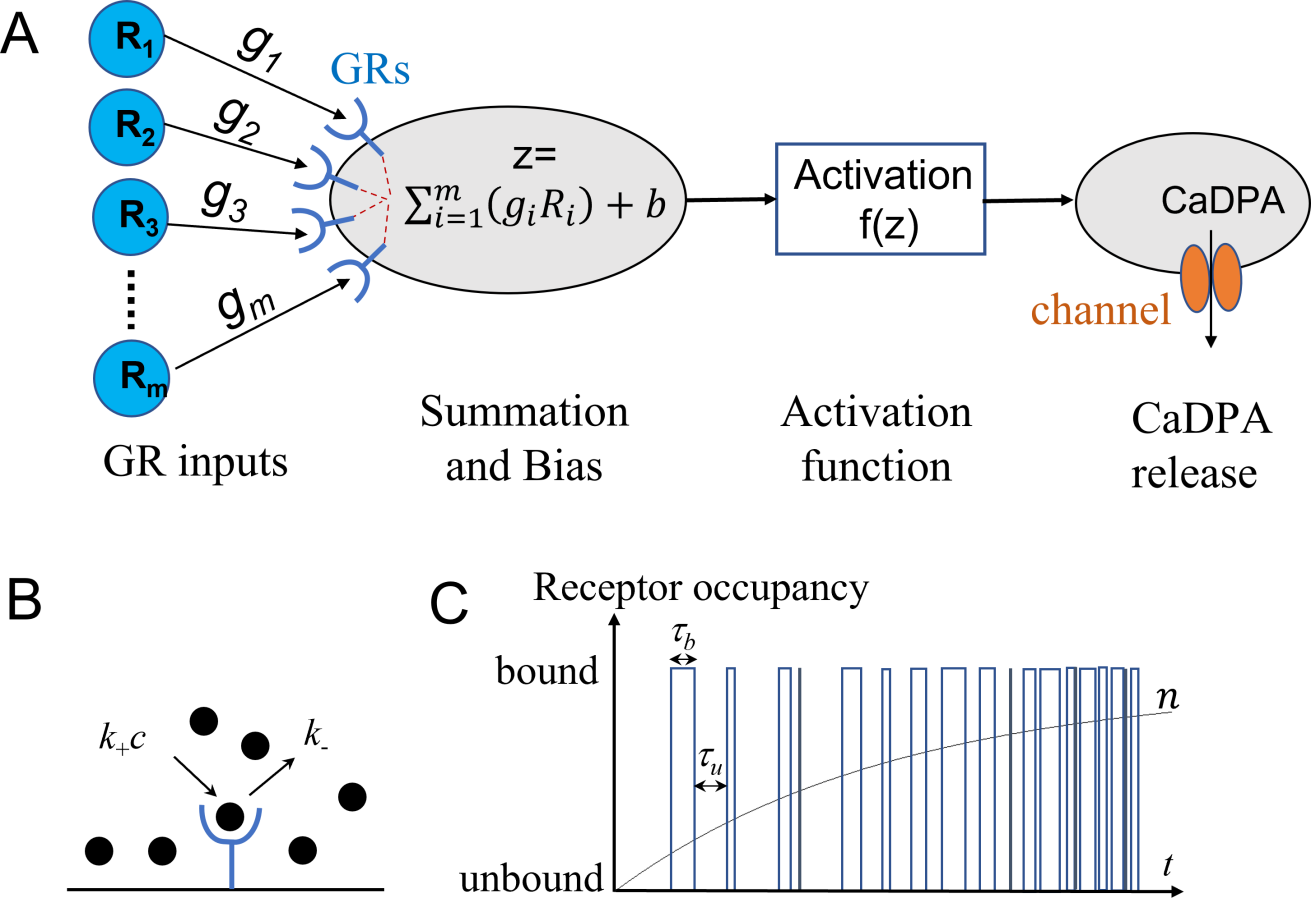
ANN-inspired model for spore germination and schematic of germinant-GR binding. (A) A spore with *m* GRs is exposed to a nutrient germinant, and each GR is activated to generate an input *R*_*i*_. These input signals are accumulated and biased to produce a trigger signal *z* above the threshold to activate the channel protein. A sigmoid function is used as the activation function *f(z*) of the channel protein. The opening of the SpoVA channel leads to the release of CaDPA molecules and completion of spore germination. (B) An unoccupied GR can bind a germinant with rate *k*_+_c, and an occupied GR can release a germinant with rate *k*_-_. (C) Binary time series of GR occupancy is depicted. *τ_b_* (=1/*k*_-_) is the average duration of bound intervals, *τ_u_* (=1/*k*_+_c) is the average duration of unbound intervals, and *n* is the fractional occupancy.

Figure 1A shows the ANN-inspired model for spore germination, in which each GR binds a germinant to generate a GR-dependent input *R*_*i*_. These signals are accumulated and biased to produce a germination trigger signal *z*, where the bias is the initial or resting value *z*_*0*_ even if input *R*_*i*_ values equal zero (Fig. S1B). Similar to the threshold mechanism described by Woese et al. (19), we assume that when the trigger signal *z* is above a critical value *z*_*c*_, the spore is committed to germinate. This commitment results in the opening of the SpoVA channel, leading to the release of CaDPA and full spore germination.

Based on the known mechanisms of spore germination and experimental findings, we make a few assumptions to describe spore germination:

1. A spore in a population has a stochastic number *m* of functional GRs following a Poisson distribution, $P\left(m\right)=\frac{{\bar{m}}^{m}}{m!}e^{-\bar{m}}$, where $\bar{m}$ is the average number of GRs per spore. Although protein number distribution can also be described by a gamma distribution and other functions, we adopted a Poisson distribution as in Woese’s model.
2. The activation of GRs by binding germinants produces a germination trigger signal *z* that can accumulate. When *z* is above a critical value *z*_*c*_, the spore commits to germinate. The value *z* is similar to the germination substance *P* generated by the activated germination “enzymes” introduced by Woese et al. (19).
3. The decision for spore’s commitment and opening of SpoVA channels for CaDPA release is controlled by the value *z* via a sigmoid activation function $f\left(z\right)=\frac{1}{1+e^{-w(z-z_{c})}}$, where *w* is the slope. The value of *f*(*z*) (between 0 and 1) can be interpreted as the cumulative probability of a spore being committed to germinate when its trigger signal is less than or equal to *z*.
4. Once a spore commits to germinate at time *t*_c_, the spore will complete rapid release of CaDPA at time *t*_*G*_ = *t*_c_ + *t*_0_, where *t*_0_ is the time between commitment and DPA release. Although many factors affect values of *t*_0_ (16, 17), we assume that *t*_0_ of committed spores is nearly constant within a spore population during germination under a given germinant concentration.

### Activation of GRs and production of a germination signal

Consider a spore containing *m* GRs in the IM. A single GR binds nutrient germinants of concentration c with rate *k*_+_*c* and unbinds germinants with rate *k_-_* (see Fig. 1B and C) (26–28). The fractional occupancy of a single GR is given by equation S2:


(1) $$n\left(t\right)=\bar{n}\left[1-e^{-\left(k_{+}c+k_{-}\right)t}\right],$$

where $\bar{n}$=$k_{+}c/(k_{+}c+k_{-})$=*c*/(*c* + *c*_0_) is the average occupancy at equilibrium and *c*_0_=$k_{-}/k_{+}$ is the characteristic concentration. The characteristic time for GR occupancy to reach equilibrium is *t*_GR_ = 1/(*k_+_c + k_-_*).

We introduce a germination signal *R* produced by GR activation, with a rate equation (19)


(2) $$\frac{dR\left(t\right)}{dt}=Kn\left(t\right)-\beta R\left(t\right),$$

where *K* is the rate constant for the production of *R* and *β* is the rate constant for R decay. Since *t*_GR_ is much smaller than the time-to-commitment *t*_*c*_, for a long exposure time (*t* >> *t*_GR_), the solution of *R* can be given by equation S4:


(3) $$R\left(t\right)=\frac{K\bar{n}}{\beta }\left(1-e^{-\beta t}\right),$$

where the steady-state value is $R_{s}=\bar{n}K/\beta$.

The germination trigger signal *z* produced by m GRs of a spore can be written as *z* $=\sum_{i=1}^{m}g_{i}R_{i}\left(t\right)+b$ as in the ANN model and is given by


(4) $$z\left(t\right)=m\bar{n}\frac{K}{\beta }\left(1-e^{-\beta t}\right)+b,$$

where we assume that each GR has the same occupancy $\bar{n}$, *g*_*i*_ is the weight factor, and each activated GR has equal weight ($g_{i}$ = 1), and *b* is the bias. The average number of activated GRs at a germinant concentration *c* is given by *m*_*a*_ = *m*$\bar{n}$, which is identical to the activated “enzymes” (19).

### Time-to-commitment and time-to-germination

When the germination trigger signal *z* is above a critical level *z*_*c*_, for example, *z*(*t*) ≥ *z*_*c*_, the spore is committed to germinate (15–17). After the commitment step, the spore will undergo release of CaDPA by opening the SpoVA channel and its replacement by water, with a lag time between commitment and CaDPA release (16, 17). Time-to-commitment *t_c_* is the time at which z is accumulated to *z*_*c*_, given by


(5) $$t_{c}=-\frac{1}{\beta }ln\left[1-\frac{m_{0}}{m\bar{n}}\right],$$

where *m*_0_ = (*z*_*c*_ – *b*)/(*K*/*β*). To initiate spore’s commitment, the activated GR number *m*_*a*_ (= *m*$\bar{n}$) of a spore must be greater than *m*_0_. Since *m* is assumed to satisfy the Poisson distribution in a spore population (19), *t*_*c*_ is heterogeneous in a spore population because of the variation in GR number *m*. Figure S2 shows the heterogeneity in *t*_*c*_ in a spore population exposed to optimal or suboptimal germinant concentrations. Spores that have more GRs commit earlier, and spores having fewer GRs commit later. For *m*$\bar{n}$ >> *m*_0_, $t_{c}\approx \frac{m_{0}}{\beta }\frac{1}{m\bar{n}}$, indicating that the time-to-commitment of a spore with a large activated GR number (*m*_*a*_) is proportional to 1/($m\bar{n}$). For a given germinant concentration, a fraction of spores that have GR number *m* less than a critical number *m*_*c*_ (= *m*_0_/$\bar{n}$) is unable to initiate commitment (*m* < *m*_*c*_). As the germinant concentration *c* is increased, the occupancy $\bar{n}$ of GRs is increased, and the fraction of spores that commit to germinate increases.

The time-to-germination *t*_*G*_ of a committed spore can be expressed as *t*_*G*_ = *t*_c_ + t_0_, where *t*_0_ is the lag time between commitment and DPA release (15–17). Experiments show *t*_0_ = ~4 min in population measurements with *B. subtilis* spores during L-alanine germination (16) and ~7.4 ± 3.0 min in measurements on multiple individual *B. subtilis* spores (17). We assume that the lag time *t*_0_ of the committed spores is nearly constant or varies in a small range in a spore population. However, factors including heat activation, germinant concentration, and average GRs/spore affect lag times between commitment and DPA release (16, 17). Consequently, the cumulative fraction of spores that germinate by time *t* after exposure to a germinant can be approximately determined by the commitment distribution by the time of *t_c_* = *t − t*_0_.

### Modeling kinetic germination distribution and spore heterogeneity

We start with a simple model using a step function for decision-making for commitment, such that the spore commits to germinate if *z*(*t*) ≥ *z*_*c*_. For a spore population, the fraction of spores with m GRs is given by a Poisson distribution *P(m*) (19). Spores that have a higher GR number (*m* ≥ *m*_*c*_) commit at different *t*_c_ and germinate at different *t*_*G*_ (= *t*_c_ + *t*_0_) for different *m*. The kinetic germination distribution *G*(*t*) is the cumulative fraction of spores that germinate by time t after the addition of germinant (with *t*_*G*_ ≤ *t*), corresponding to spores that commit by the time of *t − t*_0_ and have a GR number *m* greater than $m_{c}/[1-e^{-\beta \left(t-t_{0}\right)}]$. Thus, the kinetic germination distribution is given by


(6) $$G\left(t\right)=1-\sum_{k=0}^{m_{t}}\left(\frac{{\bar{m}}^{k}}{k!}e^{-\bar{m}}\right)$$

where $m_{t}=m_{c}/\left[1-e^{-\beta \left(t-t_{0}\right)}\right]$.

Figure S3 shows theoretical germination distributions for a spore population with different germinant concentrations. As reported by Woese et al. (19), the germination distributions show a step-like behavior due to use of a step function as the activation function and Poisson distribution of GR numbers. With a high germinant concentration, spores rapidly germinate with an optimal fraction of germination. However, with a suboptimal germinant concentration, a fraction of spores is ungerminated (Fig. S3a), and a higher average GR number shifted the germination distribution to a shorter germination time (Fig. S3b).

### Modeling kinetic germination distribution using sigmoid activation function

As the fundamental building block of the ANN, a sigmoid function *f*(*z*) is used to model the activation for the decision-making of spore’s commitment,


(7) $$f\left(z\right)=\frac{1}{1+e^{-w\left(z-z_{c}\right)}}=\frac{1}{1+e^{-w\bar{n}\left(\frac{K}{\beta }\right)\left[m\left(1-e^{-\beta t}\right)-m_{c}\right]}},$$

where *w* is the slope of the sigmoid function and *m*_*c*_ = *m*_0_/$\bar{n}$ is the critical GR number required for germination. For a spore population exposed to a constant germinant concentration, the kinetic germination distribution is given by


(8) $$G\left(t\right)=\sum_{m=0}^{\infty }\left(\frac{{\bar{m}}^{m}}{m!}e^{-\bar{m}}\frac{1}{1+e^{-w\bar{n}\left(\frac{K}{\beta }\right)\left[m\left(1-e^{-\beta \left(t-t_{0}\right)}\right)-m_{c}\right]}}\right)$$

We can easily prove that equation 8 approaches to equation 6 when slope *w* is sufficiently large such that *f*(*z*) becomes a step function, which is equal to one for *m* ≥ *m*_*t*_ or 0 for *m < m*_*c*_.

**Fig 2:**
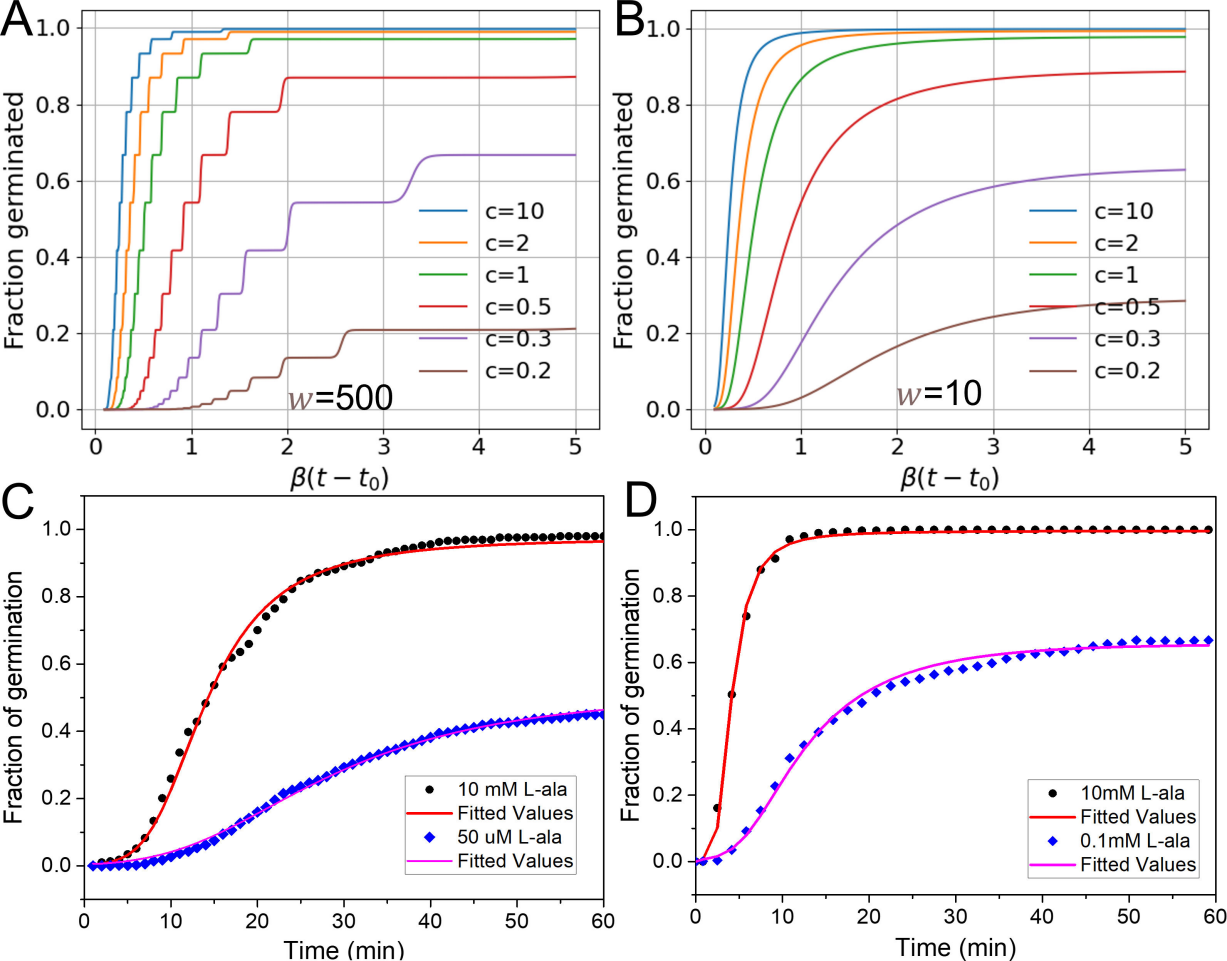
Kinetic germination distributions using a sigmoid activation function. (A) Fraction of germinated spores with slope of *w* = 500 under different germinant concentrations. (B) Fraction of germinated spores with slope of *w* = 10. The germinant concentration *c* is normalized by *c*_0_. The simulation parameters are $\bar{m}$=10, *z*_*c*_ – *b* = 1, and *K* = 0.5*β*. (C) Fitted fractions of germination of *B. subtilis* PS533 spores with L-alanine at 10 mM and 50 µM. The experimental data (scattered points) were from Zhang et al. (11) and theoretically fitted with parameters of $\bar{m}$ = 24, *w* = 5.0, *β* = 0.055 min^−1^, *K* = 0.086*β*, and *c*_0_ = 0.052 mM, with *t*_0_ = 2 min and *z*_*c*_ – *b* = 1. (D) The fitted fractions of germination of *B. cereus* spores with L-alanine of 10 and 0.1 mM. The experimental data (scattered points) were from Kong et al. (29) and fitted with parameters of $\bar{m}$ = 7, *w* = 4.5, *β* = 0.077 min^−1^, *K* = 0.75*β*, and *c*_0_ = 0.305 mM, with *t*_0_ = 1 min and *z*_*c*_ – *b* = 1.

Figure 2A and B show kinetic germination distributions for a spore population using the sigmoid activation function. With a large slope value of *w* = 500, the activation function *f*(*z*) is close to a step function, and the germination distribution curves (Fig. 2A) show a step-like behavior as in Fig. S3. However, with a small slope value of *w* = 10, the germination distribution curves become smooth (Fig. 2B). Figure 2C and D show theoretical fittings to the experimental data of kinetic germination distributions of *B. subtilis* and *Bacillus cereus* spores germinating with L-alanine, using equation 8. With an optimal germinant concentration, spores germinated with an optimal fraction of germination. With a low germinant concentration, a fraction of spores is ungerminated. The theoretical model fitted the experimental data well for both optimal and suboptimal concentrations.

### Modeling distribution in time-to-germination *t*_*G*_

Since a spore that germinates at time *t* commits to germination at *t*_c_ = *t* − *t*_0_, the distribution in time-to-germination is given by the derivative of the germination distribution *G*(*t*)


(9) $$\frac{dG\left(t\right)}{dt}=\sum_{m=0}^{\infty }\left(\frac{{\bar{m}}^{m}}{m!}e^{-\bar{m}}f\left(z\right)\left[1-f\left(z\right)\right]wKm\bar{n}e^{-\beta \left(t-t_{0}\right)}\right)$$

The fraction of spores that germinate at time *t* within a time interval Δ*t* is given by $P\left(t\right)=\frac{dG\left(t\right)}{dt}$ Δ*t*. Figure S4A shows theoretical distributions in time-to-germination *t*_*G*_ of *B. cereus* spores germinating with L-alanine of a high concentration (*c* = 50*c*_0_) and a suboptimal concentration (*c* = 0.8*c*_0_). As a comparison, Figure S4B shows the experimental distributions in *T*_release_ of *B. cereus* spores germinating with 10- and 0.1-mM L-alanine, respectively (29). The theoretical results are consistent with the experimental data for both optimal and suboptimal L-alanine concentrations.

### Modeling spore’s commitment

Spore’s commitment is an irreversible step following germinant GR binding. When exposed to a short germinant pulse with a duration *T*_*b*_, a fraction of spores with *t*_c_ ≤ *T*_*b*_ commits to germinate during the germinant pulse and the GR’s number *m* of the committed spores must be equal to or greater than $m_{T}=\frac{m_{c}}{1-e^{-\beta T_{b}}}$. Consequently, kinetic germination distribution of a spore population exposed to a short germinant pulse with concentration *c* is given by


(10) $$G\left(t\right)=\sum_{m=m_{T}}^{\infty }\left(\frac{{\bar{m}}^{m}}{m!}e^{-\bar{m}}\frac{1}{1+e^{-w\bar{n}\left(\frac{K}{\beta }\right)\left[m\left(1-e^{-\beta \left(t-t_{0}\right)}\right)-m_{c}\right]}}\right)$$

Figure S5A shows the theoretical fractions of germinated spores exposed to short germinant pulses of different durations. As a comparison, Figure S5B shows the experimental fractions of germination of PS533 spores (wild-type) with L-val with either a constant concentration or various pulse durations (17). The theoretical predictions are congruent with the experimental data.

### Modeling spore memory

Spore memory is when spores retain a response to a transient germinant exposure, making a second germinant exposure trigger more efficient germination. When a population of spores is exposed to the first germinant pulse (duration *T*_*b*_ and germinant concentration *c*), a fraction of spores with GR’s number *m* $\geq m_{T}\;\left[=\frac{m_{c}}{1-e^{-\beta T_{b}}}\right]$ generates a sufficient number of activated GRs (*m*$\bar{n}$) and produces a germination signal above the threshold *z* ≥ *z*_*c*_, so they commit to germinate as predicted by equation 10. For spores with GR’s number *m* less than *m*_*T*_, the first germinant pulse only produces a subthreshold level of activated GR’s number ($m\bar{n}$) and produces a subthreshold germination signal $R_{m1}=m\bar{n}\frac{K}{\beta }\left(1-e^{-\beta T_{b}}\right),$ and $z_{m1}=m\bar{n}\frac{K}{\beta }\left(1-e^{-\beta T_{b}}\right)+b$ (with *z*_*m*1_ < *z*_*c*_), so that they are unable to germinate by the first germinant pulse. However, after the removal of the first germinant pulse, the germination signal *R* remains with a decay rate of *β* until the arrival of the second germinant pulse. From equation 2, the germination signal *R* after the removal of the first germinant pulse is given by


(11) $$R_{m}\left(t\right)=m\bar{n}\frac{K}{\beta }\left(1-e^{-\beta T_{b}}\right)e^{-\beta \left(t-T_{b}\right)}.$$

When the second germinant pulse (with the same duration and concentration) arrives at time *t* = *T*_2_, the germination trigger signal of the ungerminated spores is given by the solution of equation 2


(12) $$z\left(t\right)=m\bar{n}\frac{K}{\beta }\left(1-e^{-\beta T_{b}}\right)e^{-\beta \left(t-T_{b}\right)}+m\bar{n}\frac{K}{\beta }\left(1-e^{-\beta \left(t-T_{2}\right)}\right)+b,$$

where the first term is the retained germination signal produced by the first germinant pulse, and the second term is the germination signal produced by the second germinant pulse. Therefore, the accumulated germination trigger signal after the second germinant pulse at *t* = *T*_2_+*T*_*b*_ is given by $z_{m2}=m\bar{n}\frac{K}{\beta }\left(1-e^{-\beta T_{b}}\right)\left(1+e^{-\beta T_{2}}\right)+b.$ Compared to the subthreshold level of germination trigger signal *z*_*m*1_ produced by the first germinant pulse, *z*_*m*2_ after the second germinant pulse is increased by a factor of $1+e^{-\beta T_{2}}$, which will cause an additional fraction of spores with $m\geq m_{T2}[=\frac{m_{T}}{1+e^{-\beta T_{2}}}]$ to commit to germination if *z*_*m*2_≥*z*_*c*_. Consequently, the kinetic germination distribution of a spore population exposed to two short germinant pulses with a separation *T*_2_ (each pulse having the same duration *T*_*b*_ and same concentration c) is given by


(13) $$G\left(t\right)=\sum_{m=m_{T}}^{\infty }\left(\frac{{\bar{m}}^{m}}{m!}e^{-\bar{m}}\frac{1}{1+e^{-w\bar{n}\left(\frac{K}{\beta }\right)\left[m\left(1-e^{-\beta \left(t-t_{0}\right)}\right)-m_{c}\right]}}\right)+\sum_{m=m_{T2}}^{m_{T}-1}\left(\frac{{\bar{m}}^{m}}{m!}e^{-\bar{m}}\frac{1}{1+e^{-w\left[z\left(t-t_{0}\right)-z_{c}\right]}}\right),$$

where *z*(*t − t*_0_) is determined by equation 12.

**Fig 3:**
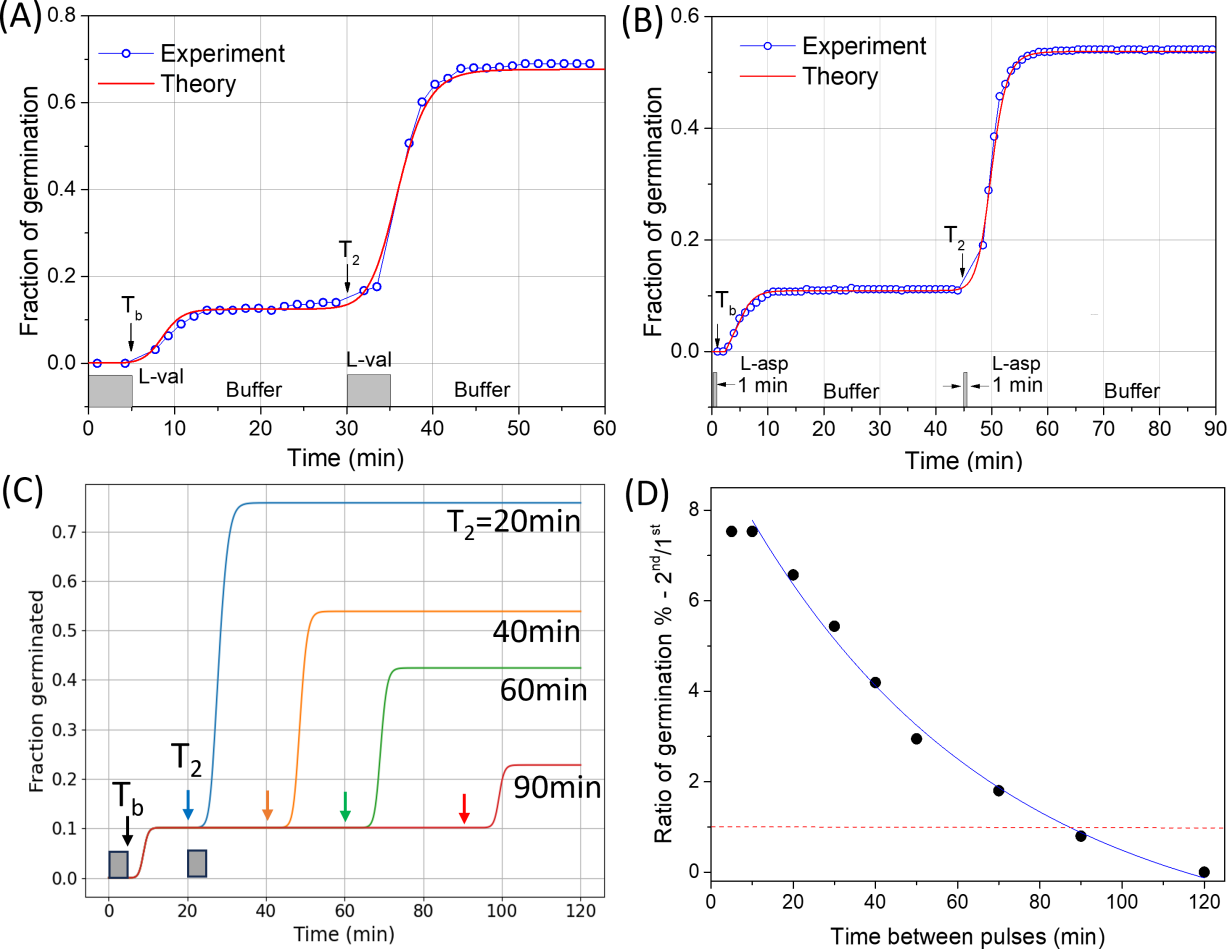
Spore memory with two germinant pulses. (A) PS832 spores (wild-type GerA level) germinated with two 5-min 3.5-mM L-val germinant pulses separated by 25 min. Experimental fraction of germination was obtained from (18) and theoretically fitted with parameters of $\bar{m}$ = 15, *β* = 0.025 min^−1^, *K*/*β* = 0.45, *w* = 4.5, *T*_*b*_ = 5 min, and *T*_2_ = 30 min. (B) PS3415 spores (high GerB* GR level) germinated with two 1-min 0.5-mM L-asn pulses separated by 45 min. Experimental data were from (17) and fitted with parameters of $\bar{m}$ = 200, *β* = 0.053 min^−1^, *K*/*β* = 0.0947, *w* = 0.8, *T*_*b*_ = 1 min, and *T*_2_ = 45 min. (C) Spore memory decays when the separation time between two germinant pulses is increased. Fraction of germinated spores exposed to two 5-min pulses as a function of time for *T*_2_ = 20, 40, 60, and 90 min, respectively. The parameters used for numerical simulation are: $\bar{m}$ = 12, *β* = 0.025 min^−1^, *K*/*β* = 0.5, *w* = 10, and *T*_b_ = 5 min. (D) Ratio of fractions of spores germinated by the second pulse and by the first pulse as the function of the separation time between two pulses (scatters). The solid line shows the exponential decay. The common parameters used for the above simulations are $\bar{n}$ = 0.94, *z*_*c*_–*b* = 1, and *t*_0_ = 4 min.

Figure 3A shows theoretical fittings to the experimental data of spore memory of *B. subtilis* PS832 spores (wild-type GerA GR levels) exposed to two 5-min 3.5-mM L-val germinant pulses (18). The theoretical model (red line) fits well to the experimental data (blue scatters). Figure 3B shows theoretical fittings to experimental data of spore memory of *B. subtilis* PS3415 spores (elevated level of GerB* GRs) with two L-asn pulse exposures. The heat-activated PS3415 spores were exposed to two 1-min L-asn pulses separated by 45 min and incubated at 37°C (17). The theoretical model (red line) fits well to experimental data (blue scatters).

Figure 3C and D presents the theoretical prediction of spore memory decay over time, given a longer interval between germinant pulses. As the separation time *T*_2_ between two pulses increases from 20 to 90 min, the fraction of germinated spores in the second pulse decreases significantly (see Fig. 3C). The ratio of spores germinated by the second pulse to those germinated by the first pulse decreases exponentially with increasing separation time (see Fig. 3D), consistent with the experimental data (see Fig. 2B of Wang et al. [18]). In particular, this ratio can drop below 1, indicating that the fraction of spores germinated by the second pulse may be lower than that germinated by the first pulse when *T*_2_ is longer. Indeed, without the memory effect, the fraction of spores germinated in response to the second pulse would be zero or lower than that of the first pulse (Fig. 3D). This is because spores with GR numbers higher than the critical threshold (*m* $\geq m_{T}$) would have already germinated during the first pulse, leaving behind ungerminated spores with lower GR numbers due to the Poisson distribution. These remaining spores receive subthreshold levels of germination trigger signal with the same germinant duration and concentration. As a result, without spore memory, they would not commit to germinate during the second pulse. These theoretical results are consistent with the experimental findings presented in (17, 18).

### Modeling kinetic release of CaDPA molecules through SpoVA channels

The efflux of CaDPA through the channel can be modeled by simple diffusion (see Supplement text). The rate equation of the intracellular CaDPA concentration C of a germinating spore can be described by equation S8


(14) $$\frac{dC}{dt}=-k_{S}f\left(z\right)\left(C-C_{out}\right),$$

where *k_S_* is the maximum diffusion rate when the channel is fully opened, *C*_out_ is CaDPA concentration outside the cell (assume *C*_out_ ~0). The solution is given by


(15) $$\frac{C\left(t\right)}{C_{0}}=exp\left\{-k_{S}\int_{0}^{t}\frac{dt^{\prime }}{1+e^{-w_{m}\left[1-e^{-\beta \left(t^{\prime }-t_{m}\right)}\right]}}\right\},$$

where *C*_0_ is the spores’ CaDPA concentration before addition of germinant, $t_{m}=-\frac{1}{\beta }ln\left[1-\frac{m_{0}}{m\bar{n}}\right]+t_{0}$ is the time-to-germination of the spores that have *m* GRs, and $w_{m}=w\left(K/\beta \right)\left(m\bar{n}-m_{0}\right)$. The kinetic CaDPA release of an individual spore is determined by three parameters *t_m_, w_m_*, and *k*_S_. The value of *k_S_* depends on the number of SpoVA channel proteins per spore (see Supplement text), which could be stochastic among individual spores in a population.

Figure S6A presents numerical simulations of kinetic CaDPA release from individual spores over time with different GR numbers for a given *k*_*S*_. The results indicate that spores with higher GR numbers germinate faster (with short *t*_*m*_), while those with lower GR numbers germinate slower. As a comparison, Figure S6B shows the experimental data of CaDPA levels of individual *B. subtilis* PS767 spores (wt GerA GRs) germinating with 10-mM L-val in 25-mM Tris–HCl buffer (pH 8.4), measured by Raman spectroscopy (30). The theoretical model predictions are consistent with the experimental data.

**Fig 4:**
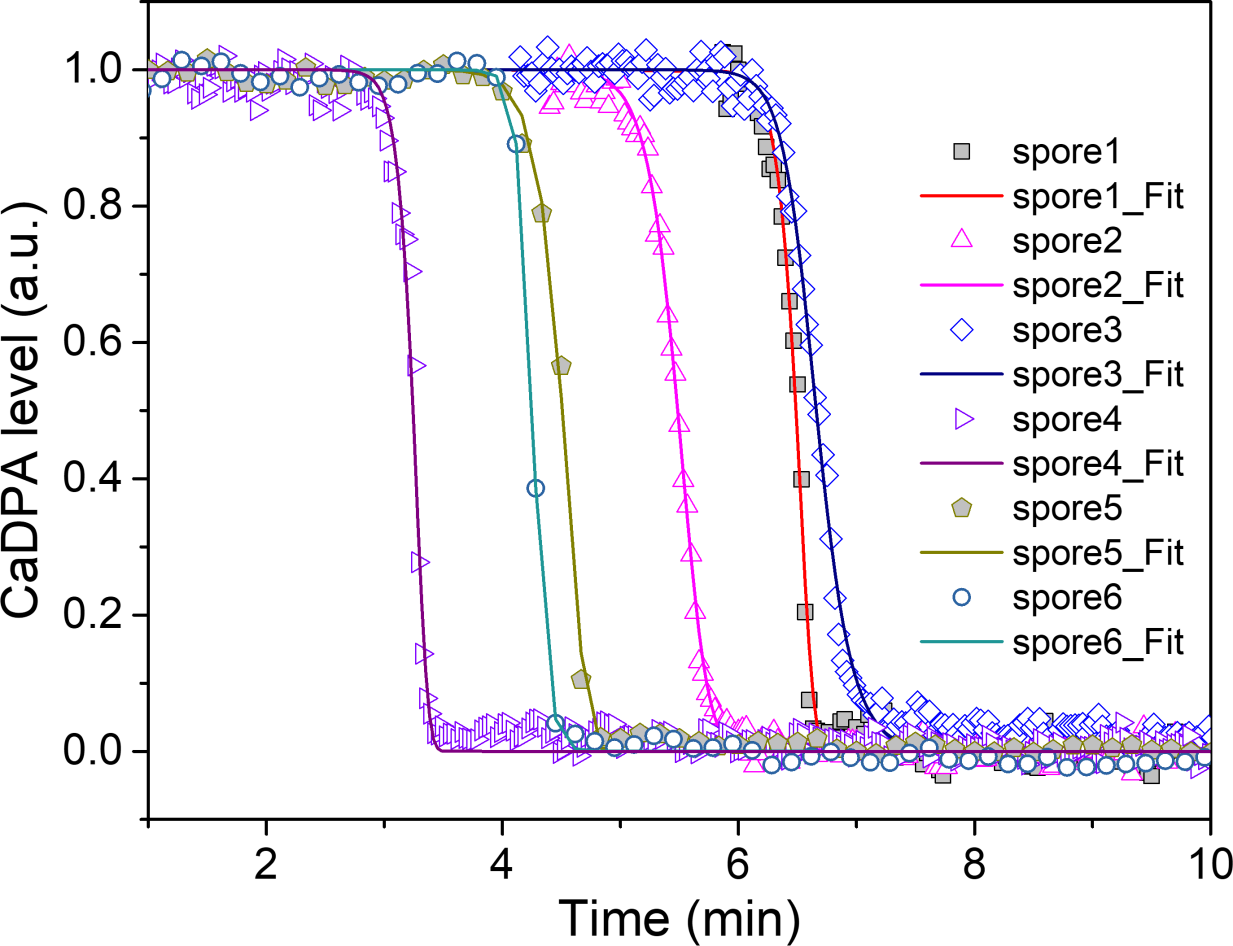
CaDPA release by multiple individual *B. cereus* spores monitored by Raman spectroscopy. *B. cereus* spores germinated with 10-mM L-alanine in 25-mM Tris–HCL buffer (pH 7.4). Raman spectra were recorded continuously, and CaDPA level data of individual spores were extracted from the intensity of the CaDPA-specific band at 1,017 cm^−1^ and normalized to its initial intensity (29). Theoretical data (line plots) are fitted to the experimental data (scatter plots) with the parameters as follows: spore1: *w*_*m*_ = 464.2, *t*_*m*_ = 6.77 min, and *K_S_* = 100.0 min^−1^; spore2: *w*_*m*_ = 319.1, *t*_*m*_ = 5.65 min, and *K_S_* = 15.1 min^−1^; spore3: *w*_*m*_ = 374.4, *t*_*m*_ = 6.60 min, and *K_S_* = 5.6 min^−1^; spore4: *w*_*m*_ = 560.1, *t*_*m*_ = 3.46 min, and *K_S_* = 100.0 min^−1^; spore5: *w*_*m*_ = 334.4, *t*_*m*_ = 4.96 min, and *K_S_* = 100.0 min^−1^; spore6: *w*_*m*_ = 772.6, *t*_*m*_ = 4.25 min, and *K_S_* = 15.2 min^−1^.

Figure 4 shows theoretical fittings to the experimental data of CaDPA release of multiple individual *B. cereus* spores monitored by Raman spectroscopy, using equation 15. The heat-activated *B. cereus* spores were germinated with 10-mM L-alanine in 25-mM Tris–HCl buffer (pH 7.4) at 37°C (29), a single spore was optically trapped, and its time-lapsed Raman spectra were recorded so that CaDPA levels of individual spores were extracted from the intensity of CaDPA-specific band at 1,017 cm^−1^ (31). The theoretical model fitted very well to the experimental data.

## DISCUSSION

This comprehensive model can account for many characteristics of spore germination, including heterogeneity, commitment, memory, and kinetics of CaDPA release by individual spores, and the model’s predictions fit well with published germination data. Our model extended Woese et al.’s simple model to explain new aspects of germination kinetics using modern ANN techniques. Each spore in a population possesses m functional GRs following a Poisson distribution. The binding of L-alanine activates a number *m*_*a*_ (=*m*$\bar{n}$) of GRs and produces a time-dependent germination trigger signal *z*, while the germinant concentration determines the average occupancy $\bar{n}$ of GRs. When *z* is above a critical value *z*_*c*_, the spore commits to germinate. While the function of trigger signal *z* is similar to the germination substance *P* in Woese’s model, the activated GRs are the biochemical mechanism of the activated germination “enzymes” (19). Although biochemical detail for signal *z* is unclear, the present model is useful in explaining how spores can confine germination to an acceptable range of conditions. For example, the time-to-commitment *t*_c_ required to accumulate the signal *z* to be above *z*_*c*_ is heterogeneous: spores with higher m GRs have shorter *t*_c_ and those with lower *m* GRs have longer *t*_c_. Consequently, time-to-germination, *t*_*G*_, exhibits heterogeneity in a spore population. Also, a “maximum” germination time is predicted from equation 5, after which no more spores can germinate, corresponding to the minimum activated GR number *m*_*a*_ greater than the critical number *m*_0_. When exposed to a suboptimal germinant concentration, occupancy $\bar{n}$ of GRs decreases, and a larger GR number *m* is required to create the minimum activated GR number, leading to the increase in the fraction of ungerminated spores. This explains the decrease in the germination percentage of a spore population with a low L-alanine concentration, but the “maximum” germination time is unchanged.

A key difference between our model and Woese et al.’s model is the introduction of the sigmoid function as the activation function, rather than the step function. The sigmoid activation function suggests that when the germination trigger signal of a spore equals to *z*, there is a cumulative probability of *f*(*z*) for the spore to commit to germinate. This widens the step-like commitment condition (with *z* ≥ *z*_*c*_). The value of slope *w* defines the window of *z* − *z*_*c*_ value for commitment, suggesting that *z*_*c*_ is probably not fixed in a spore population but varies slightly among individual spores. Thus, individual spores not only have different integer GR numbers *m* but also slightly different critical values *z*_*c*_. The strong proof of this sigmoid activation function is the disappearance of step-like behavior in kinetic germination distributions (Fig. 2B), which fits well with the experimental distributions (Fig. 2C and D), whereas the “step function” character was a major result predicted by Woese et al.’s model (19).

The present model predicts some new aspects of spore germination that can be used to test its validation and/or refine our understanding of the process. (i) It predicts that memory of spore’s response to a germinant pulse decays over time with a rate constant of *β*, so that the fraction of spores germinated by the second pulse can be lower than that germinated by the first pulse or even zero with a sufficiently long interval or with an elevated temperature for incubation between germinant pulses. With the first germinant pulse, the spore population divides into two subpopulations, one that commits to germinate and one that does not. Spores in committed subpopulation have a higher number *m* of GRs, whereas spores of uncommitted subpopulation have lower number m of GRs that cannot generate sufficient numbers of activated GRs by the first germinant pulse. Without the memory of spore’s response to the first pulse, the spores of the uncommitted subpopulation would not produce sufficient activated GRs and would not commit to germinate with the second germinant pulse. The increase in the separation time *T*_2_ or the increase in the incubation temperature between two pulses that would increase the decay rate *β* will enable the reduction of spore memory. Indeed, the existence of a “zero” fraction of spores germinated by the second germinant pulse is a direct proof of the loss of spore memory. (ii) It is predicted that kinetic germination distribution largely depends on the probability distribution of GR number m in a spore population, *P*(*m*). While a Poisson distribution is assumed for a population of wild-type spores, the modification in *P*(*m*) will change the kinetic germination distribution. Over-expressing GerA or GerB* receptors in a genetically manipulated strain may modify *P*(*m*) and thus change the germination distribution (1, 2). In addition, the exposure of a wild-type spore population (with a Poisson distribution of GRs) to a germinant pulse will divide the spores into two subpopulations, one that commits to germinate and one that does not. The uncommitted subpopulation of spores will have different GR distribution from the original *P*(*m*) and thus different kinetic germination distributions when they are germinated with a constant germinant concentration. (iii) It is predicted that the kinetics of CaDPA release of an individual spore that has m GRs is determined by *t_m_, w_m_*, and *K*_S_. Under a constant germinant concentration, the time-to-germination *t*_*G*_ is determined by *t*_*m*_, the spore with higher GRs has a shorter *t*_*G*_, and spores with fewer GRs have a longer *t*_*G*_. When *z* ≥ *z*_*c*_, the spore commits to open the SpoVA channel, while the diffusion constant is assumed to be proportional to the sigmoid activation function to account for the commitment window of the *z* − *z*_*c*_ value for opening the SpoVA channel. The rapid DPA release time Δ*T*_release_ (= *T*_release_ - T_lag_) is dominated by *w_m_* and *K*_S_. For a given *K*_S_ value, spores with more GRs have smaller Δ*T*_release_ values due to large *w*_*m*_ and spores with lower GRs have slightly larger Δ*T*_release_ values. In addition, spores with larger diffusion rate *K*_S_ may have shorter Δ*T*_release_. Incubation at a high temperature and suboptimal heat activation may reduce the diffusion constant. The analysis of kinetic CaDPA release of individual spores would allow the testing of this prediction.

The new model could also be useful in generating new data in several areas. (i) It may predict how environmental factors, including temperature, pH, and nutrient availability, affect the kinetics of spore germination. For instance, it can help determine optimal temperature ranges and germination times that maximize the speed and extent of spore germination; a food company might know the optimum time to incubate a food with a germinant to give the highest level of inactivation of spores by heat. (ii) It can be adapted to describe AGFK germination where two different GRs (GerB and GerK) are essential for germination (32). A complex of GerB/GerK receptors can be theoretically treated as a single unit of functional GRs for AGFK-GR binding and production of germination signal. This enables a deeper understanding of how interaction of two different GRs influences germination kinetics. (iii) It might be useful in assessing what germination step is altered by changes in IM fluidity. It is known that spores with different IM fluidity have very different germination rates (33, 34). The alterations in IM fluidity would change the production rate *K* and decay rate *β* of germination signal and thus influence germination rates. (iv) The model could be used to learn what germination step is altered by heat activation. It is expected that optimal heat activation may increase the number of functional GRs per spore, GR’s occupancy by the germinant, and the production rate *K* of germination signal, which will change the commitment and germination steps.

## MATERIALS AND METHODS

The *Bacillus* strains for spore germination fitting used in this work were (i) *B. subtilis* PS832, spores of which have wild-type levels of GerA; (ii) PS533 (wild-type), strain PS832 that carries plasmid pUB110 encoding resistance to kanamycin; (iii) PS3415, spores of which have elevated levels of a modified GerB, GerB* that responds to L-asn alone; (iv) PS767, spores carrying a transcriptional lacZ fusion to the promoter of the gerA operon encoding the GerA GR and retaining the wild-type gerA operon and its wild-type promoter, generated by transforming strain PS832 with plasmid AAM81; and (v) spores of *B. cereus* T. Spore preparation and germination conditions were described previously (7, 11, 17, 18).

All the simulations in this work were implemented by Python (version 3.12.5) programming. Programming of the model involved writing Python scripts to initialize model parameters, defined the activation function, and curve-fitted the experimental data for obtaining the optimal model parameters. Libraries including NumPy and Pandas were used for data manipulation and computations. The scipy.optimize library in Python was used for conducting the curve fitting of the model to the input data to generate the optimal model parameters by minimizing the error between the predicted and the experimental outcomes. Examples of Python codes used in this work are given in the supplemental material (Software S1 to S3).

## Data Availability

All data are available in the main text or the supplemental material.

## SUPPLEMENTAL MATERIAL

Supplemental material mbio.00596-25-s0001.docx Equations S1-S8, Fig. S1-S6, and Software S1-S3.

ASM does not own the copyrights to Supplemental Material that may be linked to, or accessed through, an article. The authors have granted ASM a non-exclusive, world-wide license to publish the Supplemental Material files. Please contact the corresponding author directly for reuse.
