# Supplementary material for: Modeling heterogeneity, commitment, and memory of bacterial spore germination
Source: mBio. 2025 Apr 2;16(5):e00596-25. doi: 10.1128/mbio.00596-25 (PMC12077093; doi:10.1128/mbio.00596-25)
Supplement: Supplemental material — Equations S1-S8, Fig. S1-S6, and Software S1-S3. [file mbio.00596-25-s0001.docx]

Supplementary Materials for

**Modeling heterogeneity, commitment and memory of bacterial spore germination**

William Li^1^, Steven Mednick^1^, Peter Setlow^2*^, Yong-Qing Li^3*^

^1^Marshall School of Business, University of Southern California, Los Angeles, CA 90089-0809, USA.

^2^Department of Molecular Biology and Biophysics, UConn Health, Farmington, CT 06030-3305, USA.

^3^Department of Physics, East Carolina University, Greenville, NC 27858-4353, USA.

*Corresponding author. Yong-qing Li, [liy@ecu.edu](mailto:liy@ecu.edu); Peter Setlow (AAM Fellow), [setlow@uchc.edu](mailto:setlow@uchc.edu)

**This PDF file includes:**

Supplementary Text

Eqns. S1 to S8

Figs. S1 to S6

Software S1 to S3

Data 1

Supplementary Text

**Activation of GRs by binding with germinants**

Consider a binding site for germinants, and let the fractional occupancy of the binding site be n. If the fluctuations in germinant concentration c are ignored, we can write a kinetic equation to describe fractional occupancy of a single GR as [1-3]

$\frac{dn(t)}{dt}=k_{+}c\left[ 1-n(t) \right]-k_{-}n(t)$. Eqn (S1)

The solution of GR occupancy from Eq. (S1) is

$n\left( t \right)=\bar{n}[1-e^{-\left( k_{+}c+k_{-} \right)t}]$, Eqn (S2)

where $\bar{n}$=$k_{+}c/(k_{+}c+k_{-})$=c/(c+c_0_) is the average occupancy at equilibrium and c_0_=$k_{-}/k_{+}$. *t_GR_* =1/(*k_+_c*+*k_-_*) and is the characteristic time for GR activation to reach equilibrium.

Similar to the production of germination substance in Woese et al. (1968), we introduce a germination signal R produced by GR activation, which is directly proportional to GR occupancy. The rate of production of R can be written as

$\frac{dR(t)}{dt}=Kn(t)-\beta R(t)$, Eqn (S3)

where *K* is the rate constant for the production of *R* and *β* is the rate constant for R decay. Since the time-to-germination of individual spores exposed to a constant optimal concentration of germinant is typically from a few to 10s of minutes [4-6] and spore memory is retained for 10s of minutes to hours [7, 8], we assume that R can be maintained for sufficiently long periods in germinating spores (*β* ^-1^ ~10^2^-10^3^s). However, the characteristic time *t_GR_* for GR’s activation is very short (on the order of ms or μs) such that *t_GR_* << *β* ^-1^. For a sufficiently long exposure time to a germinant (t >> *t_GR_*), the solution of Eq. (S3) can be given by:

$R\left( t \right)=\frac{K\bar{n}}{\beta}\left( 1-e^{-\beta t} \right)$, Eqn (S4)

where the value of R approaches to a steady-state level, $R_{s}=\bar{n}K/\beta$.

**Activation of multiple, independent GRs by binding with germinants.** Consider a spore containing m functional GRs in the IM, each at positions x_μ_, where μ= 1, 2, . . . , m, the kinetic equations for the occupancy of each GR are given as [1]

$\frac{dn_{\mu}(t)}{dt}=k_{+}c\left( \vec{x}_{\mu},t \right)\left[ 1-n_{\mu}\left( t \right) \right]-k_{-}n_{\mu}\left( t \right).$ Eqn (S5)

Assume that the germinant concentration c at each GR’s position is constant and the response of each GR unit is identical and independent [1]. As the exposure time t is sufficiently longer such that the occupancy of each receptor reaches equilibrium, the germination signal of the μ-th GR is R_μ_(t) $\approx K\bar{n}\left( 1-e^{-\beta t} \right)/\beta$*,* where we assume that average occupancy of each GR at equilibrium is the same as $\bar{n}$. The summed germination signal R produced by m GRs is given by

$R\left( t \right)=m\bar{n}\frac{K}{\beta}\left( 1-e^{-\beta t} \right)$. Eqn (S6)

**Opening of SpoVA channels and kinetic release of CaDPA molecules**

The probability of the SpoVA channel being open, P_O_​(t), can be modeled using a first-order differential equation:

$\frac{dP_{O}(t)}{dt}=k_{1}\left( 1-P_{O} \right)-{k_{2}P}_{O}$, Eqn (S7)

where $k_{1}$is the rate constant for the channel opening, and *k*_2_ is the rate constant for the channel closing. Assume that Eq. (S7) reaches the steady state very rapidly (a time scale in milliseconds or less), such that the open probability at equilibrium is given by $P_{O}=k_{1}/(k_{1}+k_{2})$. The permeation by molecules like Ca-DPA can be described using the Nernst-Planck equation [9]. The flux of Ca-DPA through the channel can be modeled by simple diffusion, $J=P_{O}{f(z)N}_{C}\frac{D}{L}(-\frac{dC}{dx})$, where *N_C_* is the number of SpoVA channel proteins, $\frac{dC}{dx}$ is the gradient of Ca-DPA concentration across the membrane, D is the diffusion coefficient, and L is the length of the channel, and $f\left( z \right)=\frac{1}{1+e^{-w(z-z_{c})}}$ is the sigmoid function controlled by the germination signal z. Since Ca-DPA is electrically neutral, we neglect the flux of Ca-DPA due to membrane potential. Thus, the rate equation of the intracellular Ca-DPA concentration C of a germinating spore can be described by

$\frac{dC}{dt}=-k_{S}f(z)\left( C-C_{out} \right)$, Eqn (S8)

where *k_S_*=$P_{0}N_{C}$D/L^2^ is the maximum diffusion rate when f(z) equals to 1, *C_out_* is Ca-DPA concentration outside the cell (assume *C_out_*~0). It should be noted that the *k_S_* value of an individual spore depends on the number of SpoVA channel proteins on spore’s inner membrane, which could be stochastic among individual spores in a population.

**Model fitting of kinetic germination distributions**

The experimental data of kinetic germination distributions of *B. cereus* spores germinating with L-alanine of 10mM and 0.1mM were combined into a single excel file (see Data S1). A Python code (see Software S1) was executed to load and display the experimental data. Two model parameters were fixed: t_0_ = 1 min and z_c_-b=1. The concentration of 0.1mM was normalized by c_0_=*k*_-_/*k*_+_ and expressed as c=c_1_, and the concentration of 10mM was expressed as c=100c_1_. The model software S1 automatically fits the parameters of $\bar{m}$, w, *β*, K, and c_1_. As a result of the model fitting, c_1_=0.328c_0_ was obtained, leading to c_0_=0.305 mM.

**References:**

- - - 1. Bialek W, Setayeshgar S. Physical limits to biochemical signaling, *Proc. Natl. Acad. Sci. U.S.A.* **102**, 10040-10045 (2005).
      2. Endres RG, Wingreen NS, Maximum likelihood and the single receptor, *Phys. Rev. Lett.* **103**, 158101 (2009).
      3. Mora T, Wingreen NS. Limits of sensing temporal concentration changes by single cells, *Phys. Rev. Lett.* **104**, 248101 (2010).
      4. Chen D, Huang SS, Li Y-Q, Real-time detection of kinetic germination and heterogeneity of single *Bacillus* spores by laser tweezers Raman spectroscopy. *Anal. Chem.* **78**, 6936–6941 (2006).
      5. Stringer SC, Webb MD, George SM, Pin C, Peck MW. Heterogeneity of times required for germination and outgrowth from single spores of nonproteolytic *Clostridium botulinum*. *Appl. Environ. Microbiol.* **71**, 4998-5003 (2005).
      6. Zhang PF, Garner W, Yi X, Yu J, Li Y-Q, Setlow P, Factors affecting the variability in the time between addition of nutrient germinants and rapid DPA release during germination of spores of *Bacillus* species. *J. Bacteriol*. **192**, 3608–3619 (2010).
      7. Zhang P, Liang J, Yi X, Setlow P, Li YQ, Monitoring of commitment, blocking, and continuation of nutrient germination of individual *Bacillus subtilis* spores. *J. Bacteriol*. **196**, 2443-2454 (2014).
      8. Wang S, Faeder JR, Setlow P, Li YQ. Memory of germinant stimuli in bacterial spores. *mBio*, **6**, e01859-15 (2015).
      9. Coalson RD, Kurnikova MG, “Poisson-Nernst-Planck theory of ion permeation through biological channels” in Biological Membrane Ion Channels, S.-H. Chung, O. Anderson, and V. Krishnamurthy, Eds. (Springer, 2007). pp. 449-484.

Fig. S1.


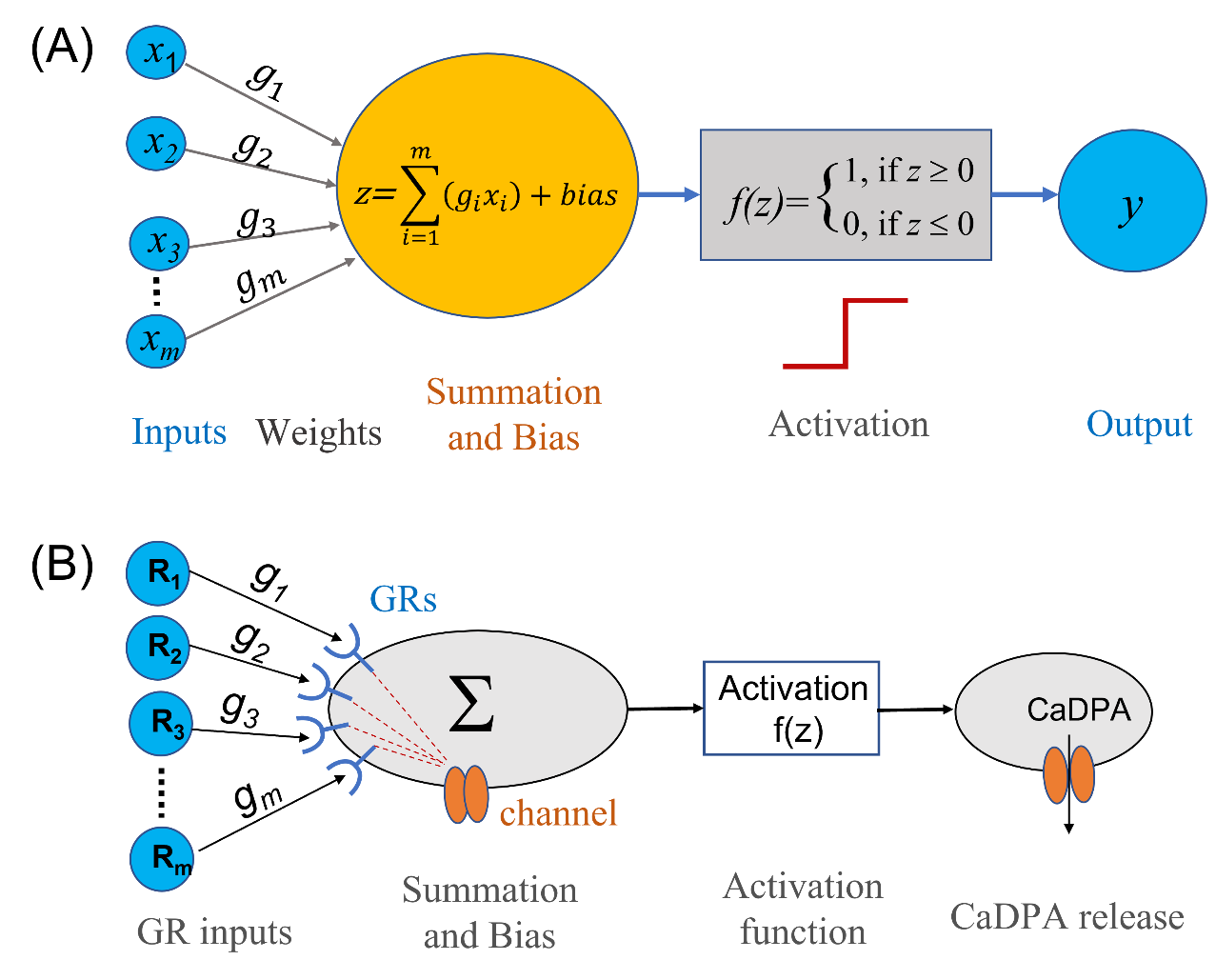


Fig. S1. ANN-inspired model for spore germination. (A) Single-layer perceptron network. Each neuron in the input layer receives an input x with subscripts i=1, 2, 3, …, m. Each input x_i_ is weighted by g_i_, and then summed and biased to send a signal z to activate a function *f(z)*. A value y between 0 and 1 is generated for output. (B) ANN model for spore germination. A spore with m GRs is exposed to a nutrient germinant, and each GR is activated to generate an input R_i_. These input signals are accumulated and biased to produce a trigger signal z above the threshold to activate the channel protein. A sigmoid function is used as the activation function *f(z)* of the channel protein. The opening of the SpoVA channel leads to the release of CaDPA molecules and completion of spore germination.

Fig. S2.

**
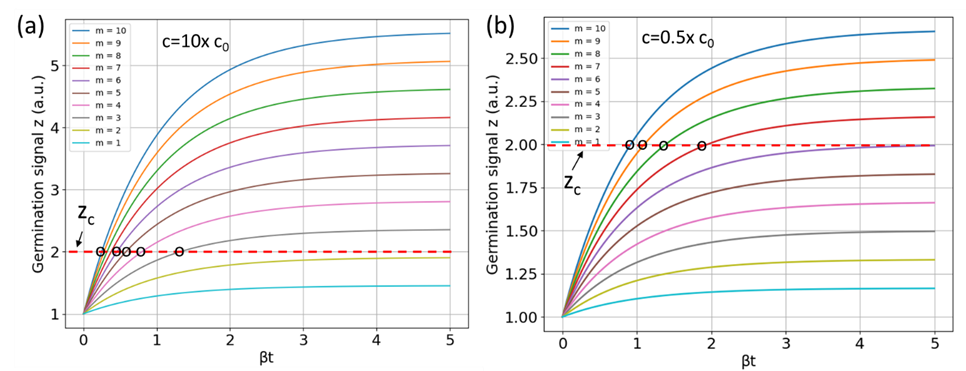
**

**Fig. S2. Heterogeneity in time-to-commitment t_C_**. The spores with different GR number m are committed to germinate at different times t_C_ after the exposure to a germinant, as the germination signal z is above the critical value z_c_. (a) Optimal germinant concentration with c=10c_0_. Spores that carry more than 2 GRs are committed. (b) Suboptimal germinant concentration with c=0.5c_0_. Spores that carry more than 7 GRs are committed and spores with fewer GRs are uncommitted. The simulation parameters are z_c_-b=1, and K=0.5*β*.

Fig. S3.


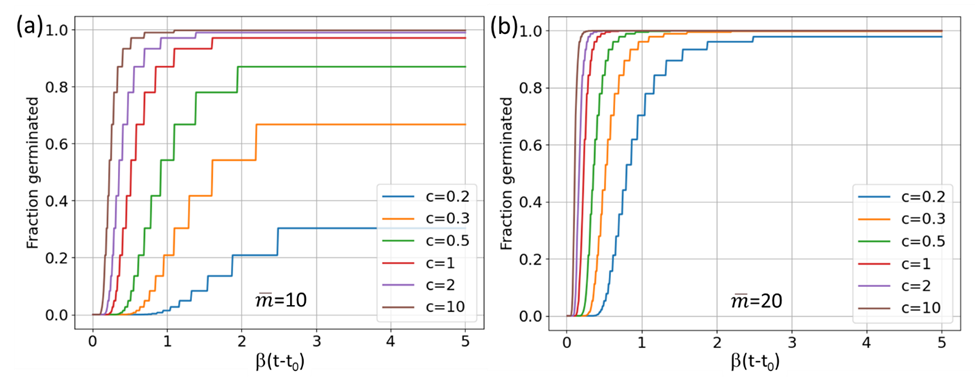


**Fig. S3**. **Germination distributions for spore populations with different germinant concentrations.** The fraction of germinated spores was plotted as the function of the exposure time. (a) The average number of GRs is $\bar{m}$=10. (b) The average number of GRs is $\bar{m}$=20. The germinant concentration c indicated in the plots is normalized by c_0_. The simulation parameters are z_c_-b=1, and K=0.5*β*.

Fig. S4.

**
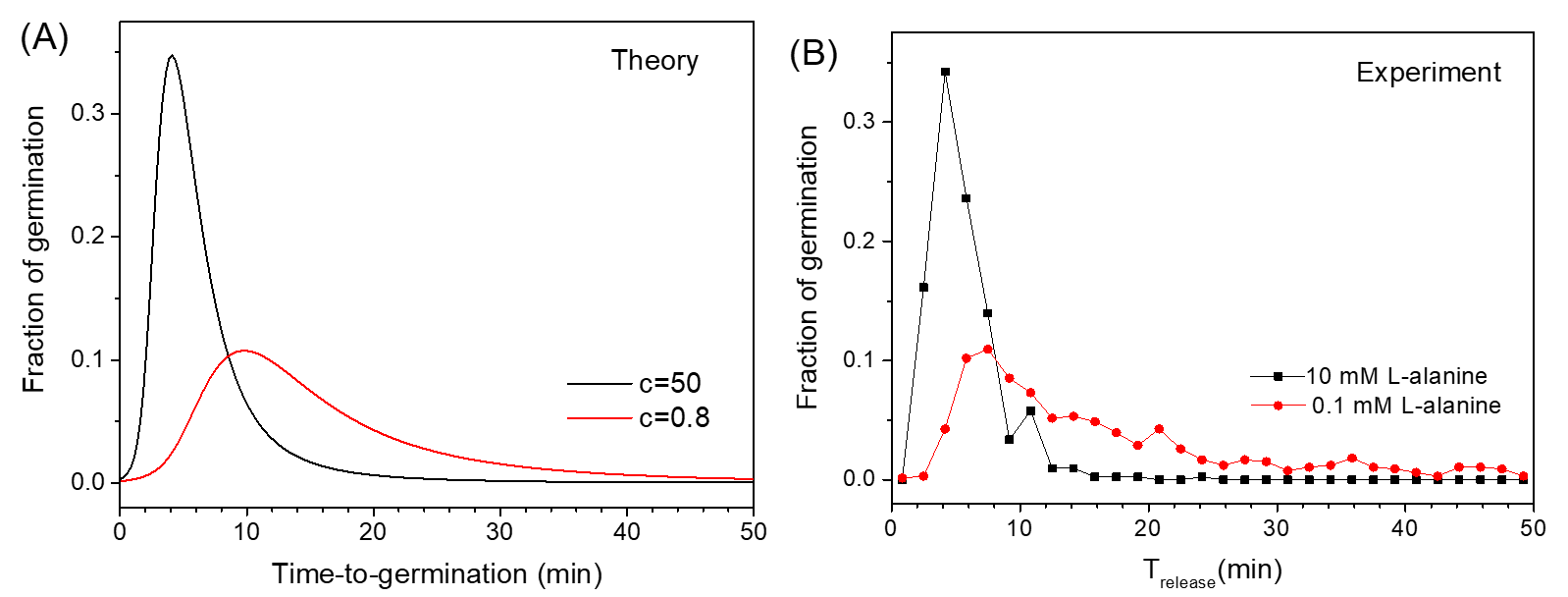
**

**Fig. S4.** **Modeling distributions in time-to-germination of *B. cereus* spores germinating with L-alanine.** (A) Theoretical fraction of germination is calculated as the function of the time-to-germination for optimal germinant concentration c=50c_0_ and sub-optimal concentration c=0.8c_0_. (B) Experimental T_release_-distribution of *B. cereus* spores germinating with L-alanine of 10 mM and 0.1 mM, obtained from [13]. The parameters used for numerical simulations in panel (A) are: $\bar{m}$ = 8, *β* = 0.06 min^-1^, K= 0.47*β*, w = 7, z_c_-b = 1, and Δt = 2 min.

Fig. S5.


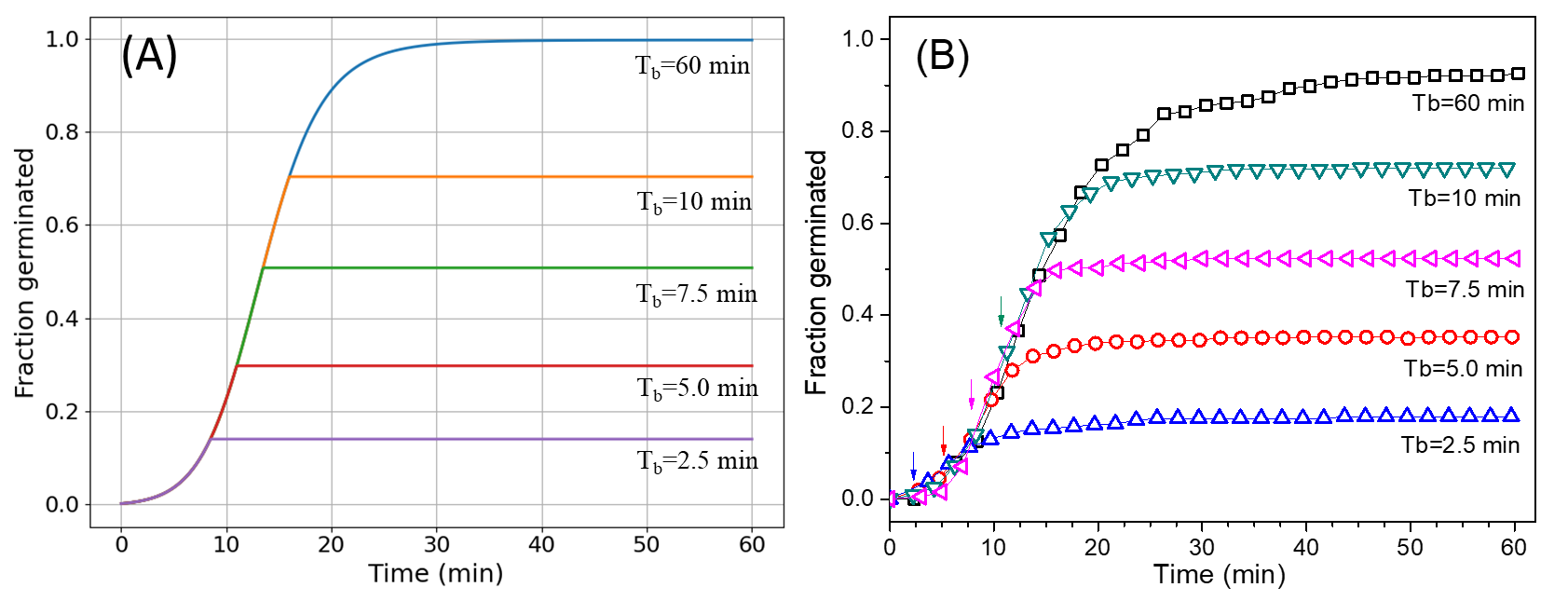


**Fig. S5.** **Modeling spore’s commitment.** (A) Theoretical fractions of germinated spores that are exposed to a short germinant pulse with different durations T_b_ of 2.5, 5.0, 7.5, 10, and 60 min, respectively. (B) Experimental fractions of germination of PS533 spores (wild type) with L-valine either with a constant concentration or with various short exposures. Heat activated spores were germinated with 10 mM L-valine either continuously (□) or for exposures of T_b_ = 2.5 min (∆), 5.0 min (○), 7.5 min (◁) or 10 min (▽) followed by blocking of further binding by removing the germinant and exposing the spores with a prewarmed mixture of 10 mM D-alanine with 25 mM K-HEPES buffer (pH 7.4) [11]. The parameters used for numerical simulations in panel (A) are: $\bar{m}$ = 8, c=50c_0_, *β* = 0.025 min^-1^, K= 0.212*β*, w = 10, z_c_-b = 0.28, and t_0_ = 6 min.

Fig. S6.


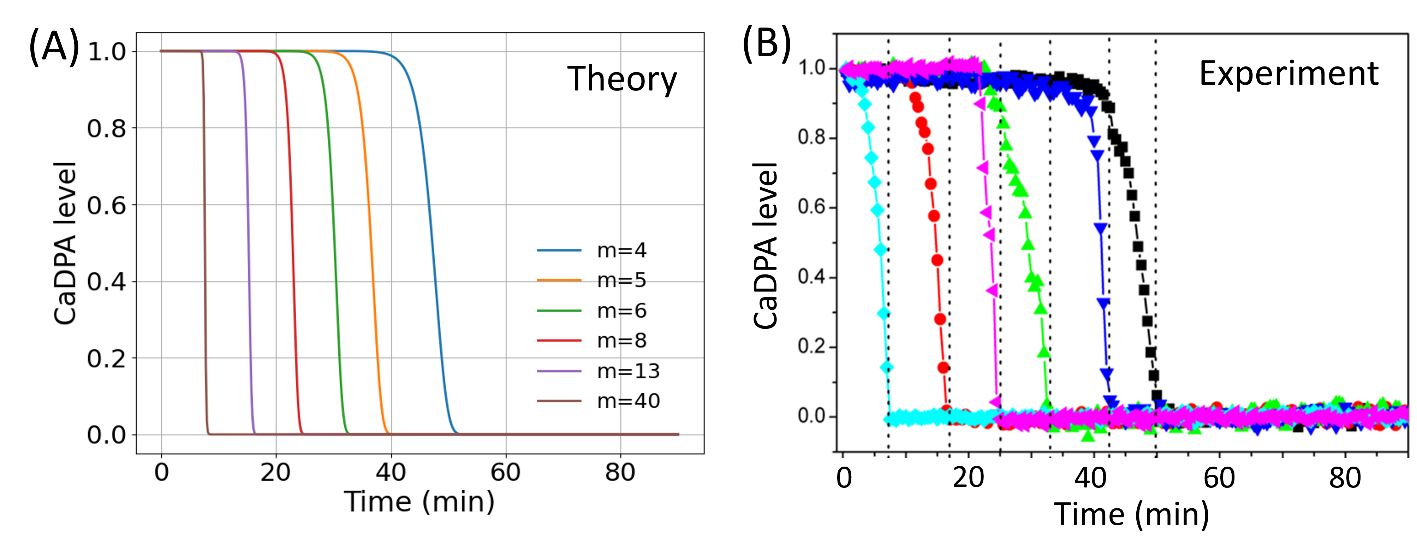


**Fig. S6.** **Kinetics of the germination of individual spores.** (A) Theoretical CaDPA level of individual spores versus time with different GRs numbers of m = 4, 5, 6, 8, 13, and 40, respectively. (B) Experimental CaDPA level of individual *B. subtilis* PS767 spores versus incubation time germinating with 10 mM L-valine in 25 mM Tris–HCl buffer (pH 8.4), measured by Raman spectroscopy [14]. The parameters used for numerical simulation in (A) are: $\bar{n}$ = 0.95, *β* = 0.015 min^-1^, K= 0.5*β*, w = 35, z_c_-b = 1, t_0_= 4 min, and *K_s_* =10 min^-1^.

**Software S1.**

**Python programming codes for Fig. 2(C, D).** This Python GUI codes is to load an experimental data containing kinetic spore germination fractions with two concentrations (Data S1 in supplementary materials), to fit the experimental data for the model parameters of $\bar{m}$, w, *β*, K, and c_1_, and to save the fitted data to a file

import pandas as pd

import numpy as np

from scipy.optimize import curve_fit

from scipy.special import factorial

import matplotlib.pyplot as plt

import os

import tkinter as tk

from tkinter import filedialog, messagebox

from matplotlib.backends.backend_tkagg import FigureCanvasTkAgg

from scipy.stats import poisson

# Fixed Parameters

t0 = 1 # t0=2 for Fig.2C

c0 = 1 # c1 was normalized by c0

b = 1 # Baseline level

zc = 2

# Define the spore germination model function with c1 and beta as parameters

def G_t(t, c1, beta, w, m, K1):

# Update c based on time

c = np.where(t > 60, c1, 100 * c1) # c1 is the concentration for 100uM

# c = np.where(t > 60, c1, 200 * c1) # for Fig.2C, c1 is the concentration for 50uM

n = c / (c + c0)

K = K1 * beta

mc = (zc - b) / (n * (K / beta))

# Adjusting for time comparison with np.where for array-based comparison

t_delayed = np.where(t < 60.1, np.maximum(t - t0, 0), np.maximum(t - 60 - t0, 0))

sum_G = 0

for m_i in range(0, 300):

# Calculate Sigmoid activation function f(z)

argument = -w * n * (K / beta) * (m_i * (1 - np.exp(-beta * t_delayed)) - mc)

f_z = 1 / (1 + np.exp(argument))

term = poisson.pmf(m_i, m) * f_z

sum_G += term

return sum_G

# Initial guesses for the parameters (c1, beta, w, m, K1)

initial_guess = [1, 0.05, 5.3, 10, 0.85]

# Bounds for the parameters c1, beta, w, m, and K1

bounds = ([0, 0, 4.5, 7, 0.75], [20, 1, 10, 30, 1]) #bounds = (c1, beta, w, m, K1)

# Function to process the files

def process_files(file_paths):

for file_path in file_paths:

try:

# Load the dataset

data = pd.read_excel(file_path)

# Check the columns

if len(data.columns) == 2:

data.columns = ['t', 'y']

elif len(data.columns) == 3:

data.columns = ['Time (t)', 'Observed Values (y)', 'Fitted Values (y_fit)']

else:

print(f'Unexpected number of columns in {file_path}')

continue

# Extract the time and observed values

t = data['t'].values if 't' in data.columns else data['Time (t)'].values

y = data['y'].values/100 if 'y' in data.columns else data['Observed Values (y)'].values

# Perform the curve fitting

try:

params, covariance = curve_fit(G_t, t, y, p0=initial_guess, maxfev=10000, bounds=bounds)

c1, beta, w, m, K1 = params

print(f'Fitted parameters: c1={c1:.3f}, beta={beta:.6f}, w={w:.3f}, m={m:.3f}, K1={K1:.3f}') # print the fit parameters

# Generate fitted values using the obtained parameters

t_fit = np.linspace(min(t), max(t), 200)

y_fit = G_t(t_fit, c1, beta, w, m, K1)

# Plot the fit

plt.plot(t_fit, y_fit, label='Fitted Model', color='red')

plt.xlabel('Time (min)')

plt.ylabel('Fraction of Germination')

plt.legend()

plt.show()

# Save fitted values to an Excel file

fit_data = pd.DataFrame({

'Time (t)': t,

'Observed Values (y)': y,

'Fitted Values (y_fit)': G_t(t, c1, beta, w, m, K1)

})

output_file_path = file_path.replace('.xlsx', '_Fitted_Line_Data.xlsx')

fit_data.to_excel(output_file_path, index=False)

# print(f'Fitted line data saved to {output_file_path}')

except RuntimeError as e:

print(f'An error occurred: {e}')

print('Trying different initial guesses...')

except Exception as e:

print(f'Failed to read {file_path} due to {e}')

# Function to open file dialog and select files

def select_files():

global selected_file_paths

selected_file_paths = filedialog.askopenfilenames(filetypes=[("Excel files", "*.xlsx")])

# Load an excel file and plot

for file_path in selected_file_paths:

if file_path:

data = pd.read_excel(file_path, sheet_name='Sheet1')

t = data.iloc[:, 0].values

y = data.iloc[:, 1].values/100

plt.scatter(t, y, color='blue', marker='o',facecolor='none', label='Exp. Data')

plt.xlabel('t (min)')

plt.ylabel('y Value')

plt.legend()

plt.show()

# Initialize the main window

root = tk.Tk()

root.title("Spore Germination Data Processor")

root.geometry("150x150")

# Create and place a button to select files

select_button = tk.Button(root, text="Select Files", command=select_files)

select_button.pack(pady=20)

# Create and place a button to process files

process_button = tk.Button(root, text="Fit Data", command=lambda: process_files(selected_file_paths))

process_button.pack(pady=20)

# Run the GUI main loop

root.mainloop()

plt.figure(figsize=(8, 6))

**Software S2.**

**Python programming codes for Fig. 3(A).** This python code is to compute and plot spore memory G(t, Tb, T2)

import numpy as np

import matplotlib.pyplot as plt

from scipy.special import factorial

from scipy.stats import poisson

# Given Parameters

m = 12 # avg m number

c = 20

beta = 0.027

K = 0.5 * beta

w = 8

c0 = 1

b = 1 # this according to your model

zc = 2 # Assuming zc = b

Tb_values = [5]

T2_values=[30]

t0=4

# Kinetic germination distribution G(t)

def G_t(t, m, c, w, Tb, T2):

n = c / (c + c0)

mc = (zc - b) / (n*(K / beta))

m_T=int(mc/(1-np.exp(-beta*Tb)))

t_delay = max(0, t-t0)

# germination with 1st pulse

sum_G = 0

for m_i in range(m_T, 200): # Summing over m from 0 to infinity (truncated at 100)

argument = -w * n * (K/beta) * (m_i * (1 - np.exp(-beta * t_delay)) - mc)

f_z= 1 / (1 + np.exp(argument))

term = poisson.pmf(m_i, m) * f_z

sum_G += term

m_T2=int(m_T/(1 + np.exp(-beta*T2)))

# Germination with 2nd pulse at t=T2

sum_G2 = 0

for m_i in range(m_T2, m_T):

argument = -w * n * (K/beta) * (m_i*(1 - np.exp(-beta * Tb))*np.exp(-beta*(t_delay-Tb)) + m_i * (1 - np.exp(-beta * (t_delay-T2))) - mc)

f_z= 1 / (1 + np.exp(argument))

term = poisson.pmf(m_i, m) * f_z

sum_G2 += term

sum_G += sum_G2

return sum_G

# Time range

t_values = np.linspace(0.01, 60, 500)

# Plot for different values of w

plt.figure(figsize=(8, 6))

for T2 in T2_values:

for Tb in Tb_values:

G_values = [G_t(t, m, c, w, Tb, T2) for t in t_values]

plt.plot(t_values, G_values, label=f'T2={T2}')

plt.title(f'G(t) vs t for w={w}')

plt.xlabel('t', fontsize=18)

plt.ylabel('Fraction germinated',fontsize=18)

# Increase the font size of the tick labels on both axes

plt.tick_params(axis='both', which='major', labelsize=16)

# Add legend with clearer font size, frame, and shadow

plt.legend(fontsize=16, markerscale=1, loc='lower right', shadow=False, frameon=True, framealpha=0, fancybox=True, borderpad=1.5)

plt.grid(True)

plt.tight_layout()

plt.show()

**Software S3.**

**Python programming codes for Fig. 4**. This Pythod code is to load the experimental data of individual spore’s CaDPA release from an excel file, to fit the model parameters (t_m_, w_m_, k_S_), and to save the fitted data to another excel file.

import numpy as np

import pandas as pd

import matplotlib.pyplot as plt

from scipy.integrate import quad

from scipy.optimize import curve_fit

from tkinter import Tk, Button, filedialog, messagebox

# Define constants

beta = 0.02 # Example value for beta

w = 30 # Example value for w

n_bar = 0.95 # Example value for n̅

zc = 2

b = 1

t0 = 4 # Example value for t0

# Define C(t)

def C(t, w_m, t_m, k_S):

def integrand(x):

return 1 / (1 + np.exp(-w_m * (1 - np.exp(-beta * (x - t_m)))))

I, _ = quad(integrand, 0, t)

return np.exp(-k_S * I)

# Function to process and fit data from selected files

def process_files(file_paths):

try:

for file_path in file_paths:

data = pd.read_excel(file_path, sheet_name='Sheet1')

t = data.iloc[:, 0].values

y = data.iloc[:, 1].values / 100

# Initial guesses for the parameters

initial_guesses = [1, 1, 1] # [w_m, t_m, k_S] = params

bounds = ([0, 0, 0], [100, 100, 100]) # [w_m, t_m, k_S] = params

try:

params, params_covariance = curve_fit(

lambda t, w_m, t_m, k_S: np.array([C(ti, w_m, t_m, k_S) for ti in t]),

t, y, p0=initial_guesses, bounds=bounds, maxfev=10000

)

w_m, t_m, k_S = params

print(f'Fitted parameters: w_m={w_m:.3f}, t_m={t_m:.3f}, k_S={k_S:.3f}')

# Compute the fitted values

y_fit = np.array([C(ti, w_m, t_m, k_S) for ti in t])

# Plot the data and the fitted curve

plt.figure(figsize=(8, 6))

plt.scatter(t, y, label='Exp. Data')

plt.plot(t, y_fit, label='Fitted Curve', color='red')

plt.xlabel('t (min)')

plt.ylabel('y Value')

plt.title(f'Spore Germination Data and Fitted Model from {file_path}')

plt.legend()

plt.show()

# Save the fitted data to a new Excel file

fit_data = pd.DataFrame({

'Time (t)': t,

'Observed Values (y)': y,

'Fitted Values (y_fit)': y_fit

})

output_file_path = file_path.replace('.xlsx', '_Fitted_Line_Data.xlsx')

fit_data.to_excel(output_file_path, index=False)

print(f'Fitted line data saved to {output_file_path}')

except RuntimeError as e:

print(f'An error occurred: {e}')

print('Trying different initial guesses...')

except Exception as e:

print(f'Failed to read {file_path} due to {e}')

# Function to open file dialog and select files

def select_files():

global selected_file_paths

selected_file_paths = filedialog.askopenfilenames(filetypes=[("Excel files", "*.xlsx")])

# Load an excel file and plot

for file_path in selected_file_paths:

if file_path:

data = pd.read_excel(file_path, sheet_name='Sheet1')

t = data.iloc[:, 0].values

y = data.iloc[:, 1].values / 100

plt.figure(figsize=(8, 6))

plt.scatter(t, y, label='Exp. Data')

plt.xlabel('t (min)')

plt.ylabel('y Value')

plt.title(f'Spore Germination Data from {file_path}')

plt.legend()

plt.show()

# Initialize the main window

root = Tk()

root.title("Spore Germination Data Processor")

root.geometry("300x200")

# Create and place a button to select files

select_button = Button(root, text="Select Files", command=select_files)

select_button.pack(pady=20)

# Create and place a button to process files

process_button = Button(root, text="Fit Data", command=lambda: process_files(selected_file_paths))

process_button.pack(pady=20)

# Run the GUI main loop

root.mainloop()

**Data S1.**

**Experimental data for Fig.2D**. These data were loaded by Software S1 to fit fractions of germination of *B. cereus* spores with L-alanine of 10 and 0.1 mM. This data should be saved as an excel file to be loaded by Software S1, in which the data of 0.1mM L-alanine was appended to the end of the data of 10mM L-alanine at t=60min for the purpose of computation.

| Time (min) | Germination Fraction (%) |
| --- | --- |
| 0 | 0 |
| 0.83333 | 0 |
| 2.5 | 16.145 |
| 4.16667 | 50.361 |
| 5.83333 | 73.976 |
| 7.5 | 87.952 |
| 9.16667 | 91.325 |
| 10.83333 | 97.108 |
| 12.5 | 98.072 |
| 14.16667 | 99.036 |
| 15.83333 | 99.277 |
| 17.5 | 99.518 |
| 19.16667 | 99.759 |
| 20.83333 | 99.759 |
| 22.5 | 99.759 |
| 24.16667 | 99.955 |
| 25.83333 | 99.955 |
| 27.5 | 99.955 |
| 29.16667 | 99.955 |
| 30.83333 | 99.955 |
| 32.5 | 99.955 |
| 34.16667 | 99.955 |
| 35.83333 | 99.955 |
| 37.5 | 99.955 |
| 39.16667 | 99.955 |
| 40.83333 | 99.955 |
| 42.5 | 99.955 |
| 44.16667 | 99.955 |
| 45.83333 | 99.955 |
| 47.5 | 99.955 |
| 49.16667 | 99.955 |
| 50.83333 | 99.955 |
| 52.5 | 99.955 |
| 54.16667 | 99.955 |
| 55.83333 | 99.955 |
| 57.5 | 99.955 |
| 59.16667 | 99.955 |
| 60 | 0 |
| 60.83333 | 0.11384 |
| 62.5 | 0.34153 |
| 64.16667 | 3.52911 |
| 65.83333 | 9.15653 |
| 67.5 | 15.35317 |
| 69.16667 | 22.72833 |
| 70.83333 | 31.19276 |
| 72.5 | 35.06339 |
| 74.16667 | 39.04787 |
| 75.83333 | 42.69082 |
| 77.5 | 45.65072 |
| 79.16667 | 47.81372 |
| 80.83333 | 51.0013 |
| 82.5 | 52.93662 |
| 84.16667 | 54.18888 |
| 85.83333 | 55.09962 |
| 87.5 | 56.35188 |
| 89.16667 | 57.4903 |
| 90.83333 | 58.05951 |
| 92.5 | 58.85641 |
| 94.16667 | 59.76715 |
| 95.83333 | 61.13325 |
| 97.5 | 61.93015 |
| 99.16667 | 62.6132 |
| 100.8333 | 63.06857 |
| 102.5 | 63.29625 |
| 104.1667 | 64.09315 |
| 105.8333 | 64.89005 |
| 107.5 | 65.5731 |
| 109.1667 | 65.80078 |
| 110.8333 | 66.01152 |
| 112.5 | 66.39457 |
| 114.1667 | 66.533 |
| 115.8333 | 66.21605 |
| 117.5 | 66.55758 |
| 119.1667 | 66.7142 |
